# Methodological issues in the creation of a diagnosis tool for dysgraphia

**DOI:** 10.1038/s41746-019-0114-0

**Published:** 2019-05-09

**Authors:** Louis Deschamps, Clement Gaffet, Saifeddine Aloui, Jerome Boutet, Vincent Brault, Etienne Labyt

**Affiliations:** 1 CEA Leti, 17 rue des Martyrs, 38054 Grenoble, France; 2 Univ. Grenoble Alpes, CNRS, Grenoble INP, Institute of Engineering Univ. Grenoble Alpes, LJK, 38000 Grenoble, France

**Keywords:** Neurodevelopmental disorders, Medical research

Asselborn et al. have created a diagnosis tool for dysgraphia, based on dynamic data acquired with common graphic tablets. The results they obtained seem very satisfactory. According to the authors, their work “can be deployed directly as a diagnostics tool”. We think that this conclusion should be qualified, as we have detected some methodological issues that we perceive to be important. In this comment, we outline three main arguments and several concerns that motivate our opinion.

First, we noticed that Asselborn et al.^1^ used different graphic tablets for both acquisitions (Wacom Intuos 3 for the D dataset, Wacom Intuos 4 for the TD dataset). As a case in point, the pressure data are widely used in this study, whereas it has been shown previously to be highly dependent on the stylus which is used.^2^ Asselborn et al. precise that “Pressure data were carefully calibrated between the two tablets”, but this is quite impossible to do, without being able to compare the values obtained with similar populations on both tablets, which was not performed in this work. Moreover, apart from this pressure calibration, there is no evidence of any method applied to ensure the independence of the results with respect to the acquisition tablet.

Second, there is a misunderstanding or omission in the definition of the TD dataset. The TD dataset is composed of 242 children from various schools around Grenoble as Asselborn et al. explain in ref. ^1^ In the “Method” part of their article, the authors wrote “None of the BHK tests from the TD dataset were rated for dysgraphia, which means that some of these children might be dysgraphic, as well”. According to the literature, we could indeed expect to find at least 5% of dysgraphic children in this dataset.^3^ The rating of our set of BHK tests recently acquired in children in primary schools confirmed the presence of 8.3% of dysgraphic children in this cohort (only considering children from second to fifth grade), according to the BHK criteria. In their introduction, Asselborn et al. themselves cited the Charles, Soppelsa, and Albarets’ study,^3^ reporting that 5–32% of children never master handwriting. But when training and testing the Random Forest models, the authors considered all children in the TD dataset as non-dysgraphic. As a consequence, the false-negative ratio of less than 1% does not make sense anymore when taking into account the dysgraphic children inside the TD dataset. Moreover, the excellent true-positive ratio of 96% claimed by the authors can also be questioned, as almost none of the dysgraphic children from the TD dataset were detected by the algorithm. In addition, considering the relatively low inter-raters correlation in the French version of the BHK test (ranging from 0.68 for beginner raters to 0.90 for very experienced ones^3^) and the intra-rater correlation (around 85%^3^), it would have been important that Asselborn et al. based their study on more than one single rater, as they aimed at creating a human independent diagnosis tool of dysgraphia.

Third, in their data-processing approach, the authors did not take into consideration the evolution of the features with the age of the subject. Handwriting evolves as the child grows, and some features do not apply the same for young children and their older counterparts. Consequently, their dysgraphia diagnosis algorithm should not be robust against children age, as the time evolution of handwriting parameters is not taken into consideration.

In addition, several remarks can be made about the Asselborn’s study.^1^ We noticed some anomalies in the acquisition process, as for example, a short stroke visible at the beginning of more than 80% of records of the children in the D dataset. This short stroke consisted of a test done by the experimenter to check the acquisition process before giving the pen to the children. This anomaly was not observable in the TD dataset, the experimenter being different for these children recorded in schools. This kind of anomaly in the data has typically to be taken into consideration before extracting features used for the machine-learning algorithm. Indeed, as this irregularity is only visible for children in the D dataset, it can artificially increase some features for most of the dysgraphic children used to create the Random Forest model, and therefore skew the final model. It appears that the authors did not exclude from the data this short “artificial” stroke before processing the dataset. For illustration, we calculated the mean in-air time ratio (see Fig. 2 in Asselborn’s paper) of the D dataset with or without correction of the anomaly. This feature was mentioned by the authors as interesting and discriminative, according to Rosenblum et al.^4,5^ We compared it with the value of 61% given in Ref. ^1^ We found out that 61% of the in-air time ratio corresponded to what we obtained without correcting the in-air time artifact at the beginning of the records. When correcting it, we found 59% of in-air time ratio. For the TD dataset, we applied some corrections as well and found an in-air time ratio of 55%. We show the histograms of the in-air time ratio before and after applying the correction in Figs 1 and 2. We can clearly see that the difference between the D and TD datasets is less important after the correction, even if this one is still statistically significant (the *p*-value given by Welch’s *t* test for mean comparison goes from 1·10^−6^ to 0.017 after the correction is applied). Considering the lack of calibration between the two acquisition tablets, other flaws like the one we just described have to be considered. This leads us to be cautious about the conclusions from Asselborn’s study.

**Fig. 1:**
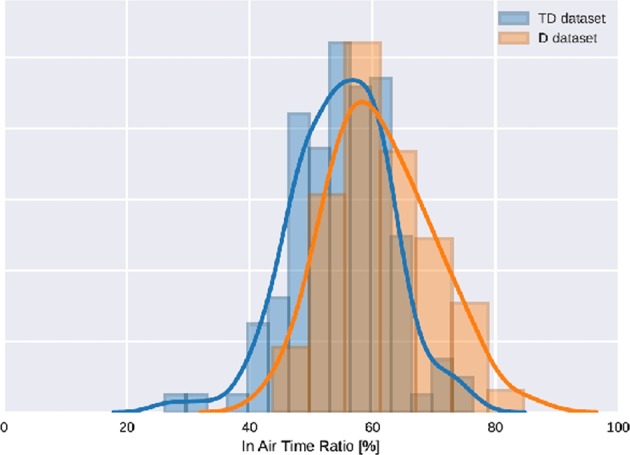
In-air time ratio before artifact correction. Histograms of the in-air time ratio without correcting the artifact are shown for D and TD datasets

**Fig. 2:**
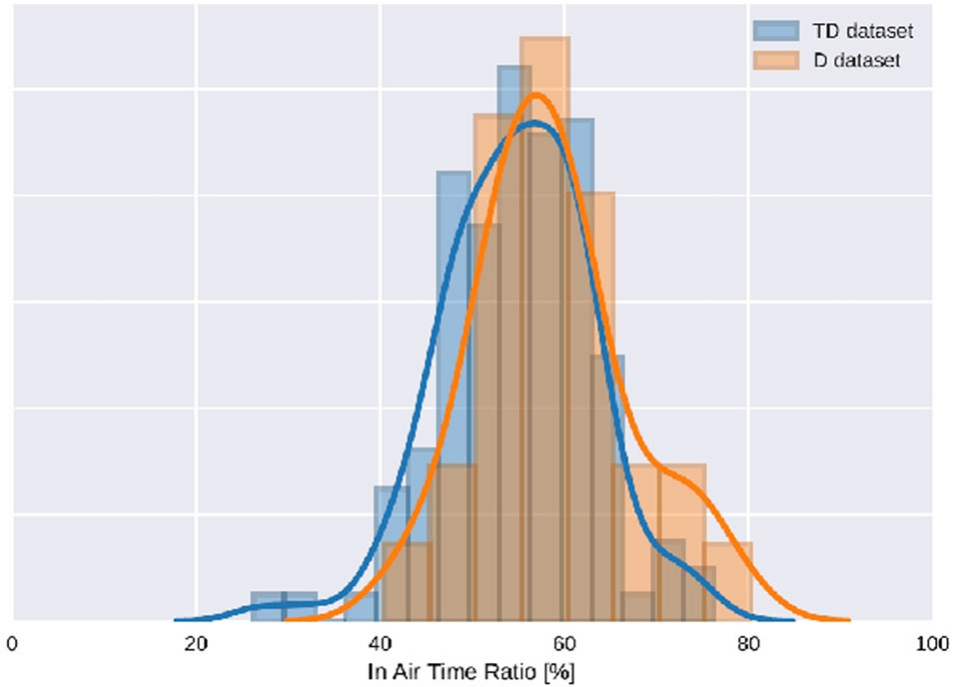
In-air time ratio after artifact correction. Histograms of the in-air time ratio after correcting the artifact are shown for D and TD datasets

Furthermore, all the dysgraphic children used in Asselborn’s study (D dataset) were recruited at the Reference Center for Language and Learning Disorders of the Grenoble Hospital (Centre Referent des Troubles du Langage et des Apprentissages, CRTLA, Centre-Hospitalier-Universitaire Grenoble), whereas all the typically developing children were recruited in schools around Grenoble. This ensures that the selected children in each group are categorized with a high confidence, notably for the dysgraphic children. However, we would like to emphasize that the dysgraphic children from the Grenoble hospital are not representative of the whole dysgraphic population in France. Indeed, children going to the CRTLA usually present more severe dysgraphia than the average. The writing performances of the children in the D dataset may therefore be worse than those of the most of dysgraphic children. This assumption is supported by the calculation of the average BHK and speed scores of the D dataset, and the comparison with a database of 23 dysgraphic children recently acquired in schools (see the “Methods” section). These 23 children come from our database acquired in primary schools near Grenoble, whose BHK texts have been rated. We found that the average BHK score of the D dataset is −3.3, whereas the average BHK score of the 23 dysgraphic children from schools is −2.6. The difference is even bigger for the speed score. The average speed score of the D dataset is –1.2, whereas it is 0.1 for the 23 dysgraphic children from schools. According to Welch’s t test, these means are significantly different (*p* *<* 0.01 and *p* *<* 0.005, respectively), thus confirming that the BHK and speed scores from the D dataset are worse than those of dysgraphic children from schools. Combining this result with the omission of the possible presence of dysgraphic children in the TD dataset suggests that the model created by Asselborn et al. is more biased toward recognizing only the most severe cases of dysgraphic children. Therefore, we think it requires to be tested at a larger scale, with dysgraphic children outside the hospital, before claiming that it could be deployed as a diagnosis tool of dysgraphia.

To conclude, given the issues raised in this comment, we would like to insist on the fact that the model performances and conclusions of the article^1^ by Asselborn et al. should be considered cautiously. Their approach is an interesting novel way of pre-diagnosing dysgraphia, but the tool they created should be tested at a larger scale, and some biases should be dealt with before it can be deployed as a diagnosis tool.

## Methods

In this paper, we processed the database used by Asselborn et al. (D and TD datasets). In addition, the average BHK score of the dysgraphic children from schools calculated in this comment is based on a large-scale study led by CEA-Leti, LPNC, and LJK. This study was conducted in accordance with the Helsinki Declaration. It was approved by the Grenoble University Ethics Committee (Agreement No. 2016-01-05-79). The writing consent of all children’s parents and the oral consent of all children have been acquired.

In total, 509 children were recruited in primary schools from various places of Grenoble suburbs (France). BHK was rated by two expert raters in order to be as objective as possible.

Handwriting acquisitions were performed on Wacom Intuos graphic tablets. Detailed information of participants and the results obtained will be presented in an article currently in preparation.

## Data Availability

For both databases, the consent of the parents to publish the database has not been acquired, and according to the EU-GDPR (European Union Global Data Protection Regulation), we cannot share the data from this study.

## Code availability

Software has been developed under C++ for the data acquisition in schools. Data processing (in air time ratio computation) and statistical analysis used standard functions under python. We do not give access to our acquisition software and codes outside of an established research collaboration framework.
